# Vertical and temporal flight patterns of coffee berry borer (Coleoptera: Curculionidae) in Hawaii

**DOI:** 10.1093/ee/nvae051

**Published:** 2024-05-29

**Authors:** Melissa A Johnson, Colby T Maeda, Ishakh Pulakkatu-Thodi

**Affiliations:** United States Department of Agriculture—Agricultural Research Service, Daniel K. Inouye US Pacific Basin Agricultural Research Center, Hilo, HI, USA; United States Department of Agriculture—Agricultural Research Service, Daniel K. Inouye US Pacific Basin Agricultural Research Center, Hilo, HI, USA; United States Department of Agriculture—Agricultural Research Service, Daniel K. Inouye US Pacific Basin Agricultural Research Center, Hilo, HI, USA; NSF Center for Integrated Pest Management, College of Agriculture and Life Sciences, North Carolina State University, Raleigh, NC, USA

**Keywords:** crop infestation, integrated pest management, phenology, trap catch, weather

## Abstract

Coffee berry borer (*Hypothenemus hampei* Ferrari) (Coleoptera: Curculionidae) is the most damaging insect pest of coffee worldwide, causing significant losses in coffee yields and quality. Knowledge of vertical and temporal flight patterns in coffee berry borer could be used to optimize spray timing and precision targeting of areas within the coffee tree, which may be more susceptible. In the present study, we estimated the vertical distribution of coffee berry borer females using traps set at 1-m intervals up to 5 m in height. We also quantified coffee berry borer infestation in the low, mid, and high canopy and documented fruit availability. Temporal flight patterns were estimated using timer traps, and correlation analyses were conducted to determine the relationship between the timing of daily flight and weather variables. Across the 4 study sites, we observed that 77%–84% of the trap catch was at 1 m, 11%–20% was at 2 m, and 1%–4% was at 3–5 m in height. Fruit infestation was significantly higher in the low branches (35%) relative to the high branches (17%). Flight height remained the same year-round, regardless of fruit availability. Coffee berry borer flew in low numbers during the day and night but peaked from 12 to 4 PM. Daily flight was positively correlated with an increase in air temperature and wind speed and negatively correlated with relative humidity. Findings from this study suggest that pesticide sprays should target low- to mid-level branches at 1–2 m in height and aim to be conducted in the early afternoon when coffee berry borer are actively flying and most vulnerable to chemical controls.

## Introduction

Coffee berry borer, *Hypothenemus hampei* (Ferrari) (Coleoptera: Curculionidae: Scolytinae), is the most damaging insect pest of coffee worldwide, causing more than $500 million in annual crop losses (Vega et al. 2015). The female coffee berry borer initiates infestation when she bores an entrance hole into the coffee fruit (“berry”) and builds galleries for reproduction in the seed (“bean”). The developing larvae feed on the endosperm tissue, causing further damage to the bean and resulting in reduced quality and yields (Duque and Baker 2003, Vega et al. 2015). The life cycle occurs almost entirely within the coffee berry, making control of this pest difficult. Male and female siblings mate within their natal berry, the males die, and mated females leave in search of a new berry to infest (Damon 2000, Vega et al. 2015). It is during this period of dispersal that adult female coffee berry borer are most vulnerable to chemical and biological controls (Jaramillo et al. 2006, Aristizábal et al. 2016, 2017).

Hawaii was one of the last coffee-growing regions in the world without an established population of coffee berry borer until it was detected in the Kona district of Hawaiʻi Island in September 2010 (Burbano et al. 2011). The tiny beetle rapidly spread across Hawaiʻi Island and was later confirmed on the neighboring islands of Oʻahu (2014), Mauʻi (2016), Kauaʻi (2020), and Lānaʻi (2020) (Gillett et al. 2020). The arrival of this global pest changed Hawaii’s coffee industry forever. Without a suite of integrated pest management (IPM) practices, including multiple sprays of the entomopathogenic fungus *Beauveria bassiana* (Hypocreales: Clavicipitaceae), frequent and efficient harvesting, and postharvest field sanitation, coffee berry borer infestation can reach as high as 95% (Johnson and Manoukis 2020). There are more than 1,000 coffee farms in the State of Hawaii, most of which are small, family-run operations that rely on manual labor to harvest the coffee and implement management practices (Stewart et al. 2018). Optimizing pest management strategies while minimizing costs is critical to the longevity of these farms, as Hawaii has some of the highest labor and production costs of any coffee-growing region (Woodill et al. 2014, Lee et al. 2023).

Many insects rely on flight for a variety of biological and ecological functions, including the location of mates, food resources, and new hosts for colonization and reproduction. The timing and height at which coffee berry borer fly may provide insight into the beetle’s interaction with the host coffee plants, as well as external triggers for flight, including weather and tree phenology. Several studies that have documented flight height in various species of ambrosia beetles have reported that beetles flying near the ground at ~0.5 m tend to attack the trunk and lower branches of the tree (Ranger et al. 2010, Hanula et al. 2011, Brar et al. 2012), while those captured at ~2.5 m above the ground prefer higher branches (Carrillo et al. 2016, Dodge et al. 2017). The timing of daily flight is likely influenced by a variety of interacting abiotic (e.g. temperature, wind speed, solar radiation, rainfall) and biotic (e.g. resource availability) factors. Earlier studies examining daily flight in ambrosia beetles have reported differing results in terms of suitable temperatures, wind speeds, relative humidity, and light intensity for flight (Gaylord et al. 2008, Kendra et al. 2012, Chen and Seybold 2014, Johnson et al. 2016, Menocal et al. 2018), suggesting that there are species-specific patterns in the host-seeking behavior of these beetles.

Some of the earliest studies of coffee berry borer flight reported captures up to 5 m in height (Leroy 1936) or higher with strong gusts of wind (Bemelmans 1930). Later studies suggested that coffee berry borer flight was concentrated at lower heights, with Dufour and Frerot (2008) reporting peak captures at 1.2 m and Uemura-Lima et al. (2010) reporting the highest captures at 0.5 m. Coffee berry borer has also been observed to fly at varying times depending on the location of the study, with peak flight times reported as early afternoon (1:30–3:30 PM, Nicaragua; Borbón-Martinez et al. 2000), late afternoon (4:00–5:00 PM, Democratic Republic of Congo; Leroy 1936), and early evening (4:00–6:00PM, Java; Leefmans 1923). Most of these studies, however, were based on observations of coffee berry borer emergence from a small number of berries and were further limited across space (single regions or farms) and time (only part of the growing season).

In the current study, we used alcohol-baited traps to examine the flight height and daily flight activity of coffee berry borer at 4 commercial coffee farms on Hawaiʻi Island. Weather variables thought to be important for insect flight (temperature, relative humidity, and wind speed) were monitored at each site, as well as fruit availability and infestation in the low, mid, and high canopy. We collected data over a full season to detect any differences in flight that may be associated with changing weather and plant phenology. Our findings will aid growers in optimizing the placement of traps for monitoring, as well as the timing and location of pesticide sprays for control. We expect that the information presented here on coffee berry borer flight patterns and associated biotic and abiotic variables will be useful for developing IPM strategies in Hawaii as well as other coffee-growing regions around the world.

## Materials and Methods

### Flight Height

The vertical distribution of dispersing coffee berry borer females was investigated in 4 commercial coffee farms on Hawaii Island from 2020 to 2021. One farm was on the east side of the island in the Hilo district at 198 m, 1 on the west side in the Kona district at 454 m, and 2 on the south side of the island in the Ka’u district at 279 and 484 m. *Coffea arabica* var. *typica* was planted at 3 farms, while 1 farm had *Coffea arabica* var. *caturra* planted. The average tree height varied from 2.2 to 3.5 m tall, and the tree age was 20–30 yr old. All farms were actively managed for coffee production, with practices including pruning, weed control, fertilization, harvesting, sanitation, and biopesticide applications of *B. bassiana* for coffee berry borer control.

**Fig. 1.**
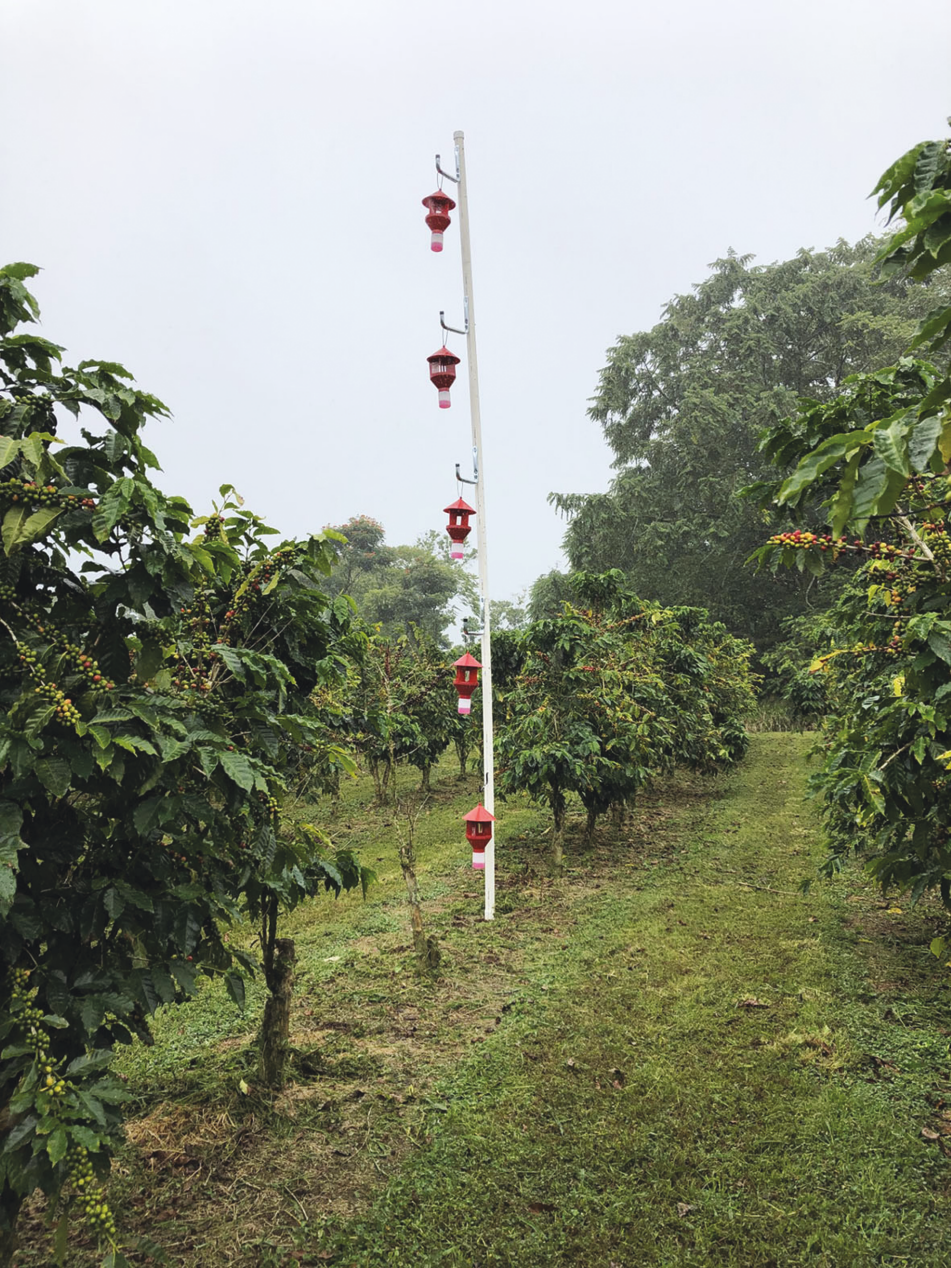
The trap pole is made from a PVC pipe, with red funnel traps placed at 1-m intervals up to 5 m in height. Traps were collected biweekly using a modified fruit picker.

Two trap poles were constructed on each farm using 1.5 m tall t-posts driven into the ground to provide stability, and a PVC pipe slid over the top (Fig. 1). Each pole was positioned randomly within a row of productive coffee and approximately 2 m away from the nearest tree. On each pole, 5 red funnel traps (Brocap traps, CIRAD, Montpellier, France) were hung from hooks 1, 2, 3, 4, and 5 m above the ground with a vertical distance of 1 m between traps (Fig. 1). Traps were baited with a 3:1 methanol:ethanol lure (40 ml) placed in a semipermeable bag (2 Mil, 3 × 4 inch) with an elution rate of ~186 mg/day (Borbón-Martinez et al. 2000) and equipped with a bottle of kill solution comprised of 200 ml of propylene glycol (Better Glycol, Better World Manufacturing Inc., Fresno, CA, USA) and 800 ml of water. Trap contents were collected in 70% ethanol on a biweekly basis at each site. The contents were sorted (coffee berry borer vs. other beetles/insects) and counted under a stereomicroscope (Leica, Microsystems GmbH, Wetzlar, Germany) (see Johnson et al. 2018 for additional details on trap collection and processing). Trap lures and kill solution were refreshed as needed during trap collection.

### Fruit Phenology and Infestation

Fruit phenology was recorded biweekly at the 4 farms where flight data was collected. Within each coffee farm, 25 trees were haphazardly selected throughout the field. From each tree, a single branch at chest height was randomly selected to score phenology. The total number of infestable green fruits (pea size and larger) was tallied for each branch, and the average was calculated across the 25 trees for each sampling date/farm. Infestation sampling followed the methods developed by Bustillo et al. (1998) and modified in Johnson et al. (2018). On 3 of the 4 farms, 15 trees located in the same plot as the trap poles were randomly selected for infestation assessments. A single branch with at least ~30 green berries was randomly selected from 3 heights (low = 1 m, mid = 2 m, and high = 3 m), and the number of green fruits that were pea size and larger were counted. Green fruits were then examined for a coffee berry borer entrance hole in the central disc, and the number of infested berries per branch was recorded. Percent infestation was calculated by dividing the total number of infested berries by the total number of green berries per branch and multiplying by 100. The average infestation (%) for each tree section (low, mid, and high) was calculated across the 15 trees for each farm/sampling date.

### Daily Flight Periodicity

**Fig. 2.**
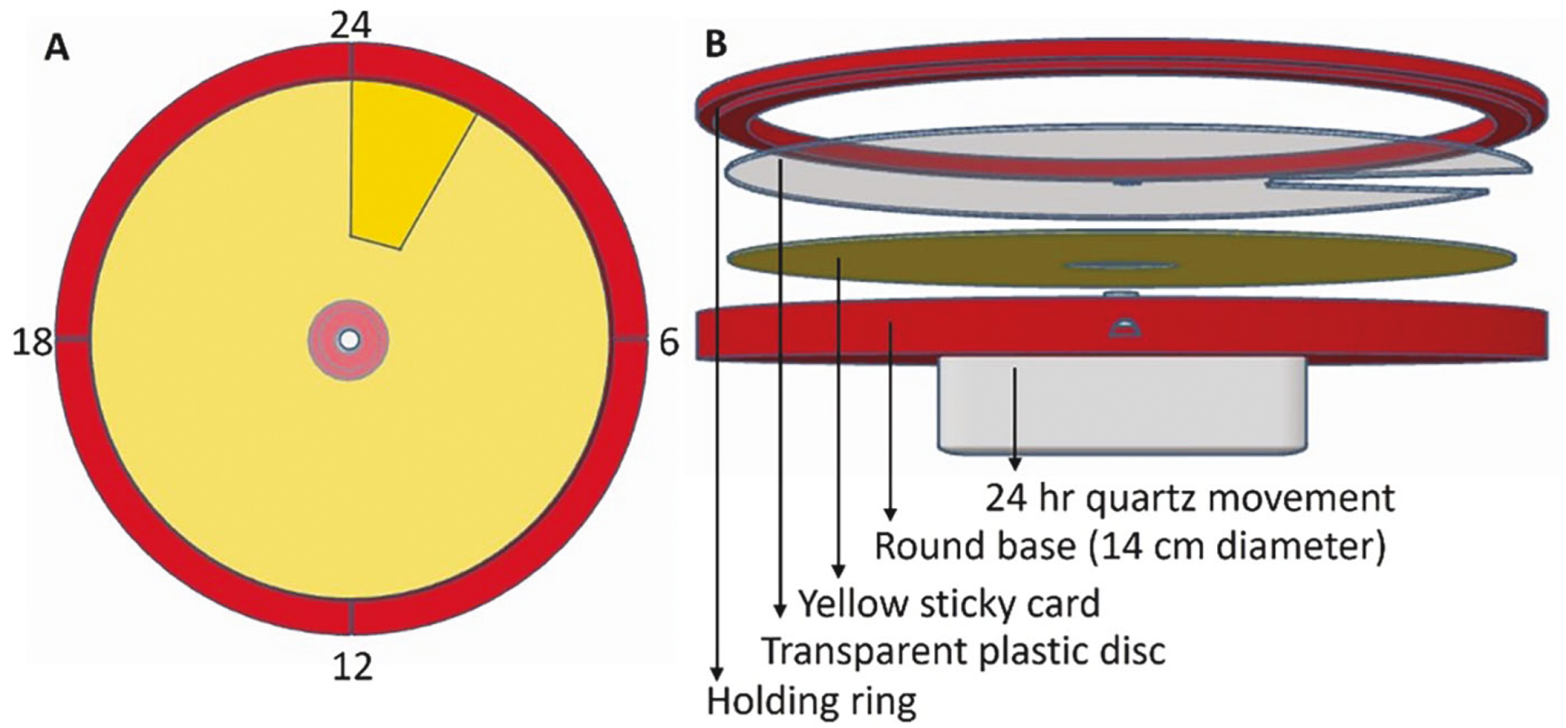
Top view A) and exploded side view B) that shows various components of the flight periodicity trap.

**Fig. 3.**
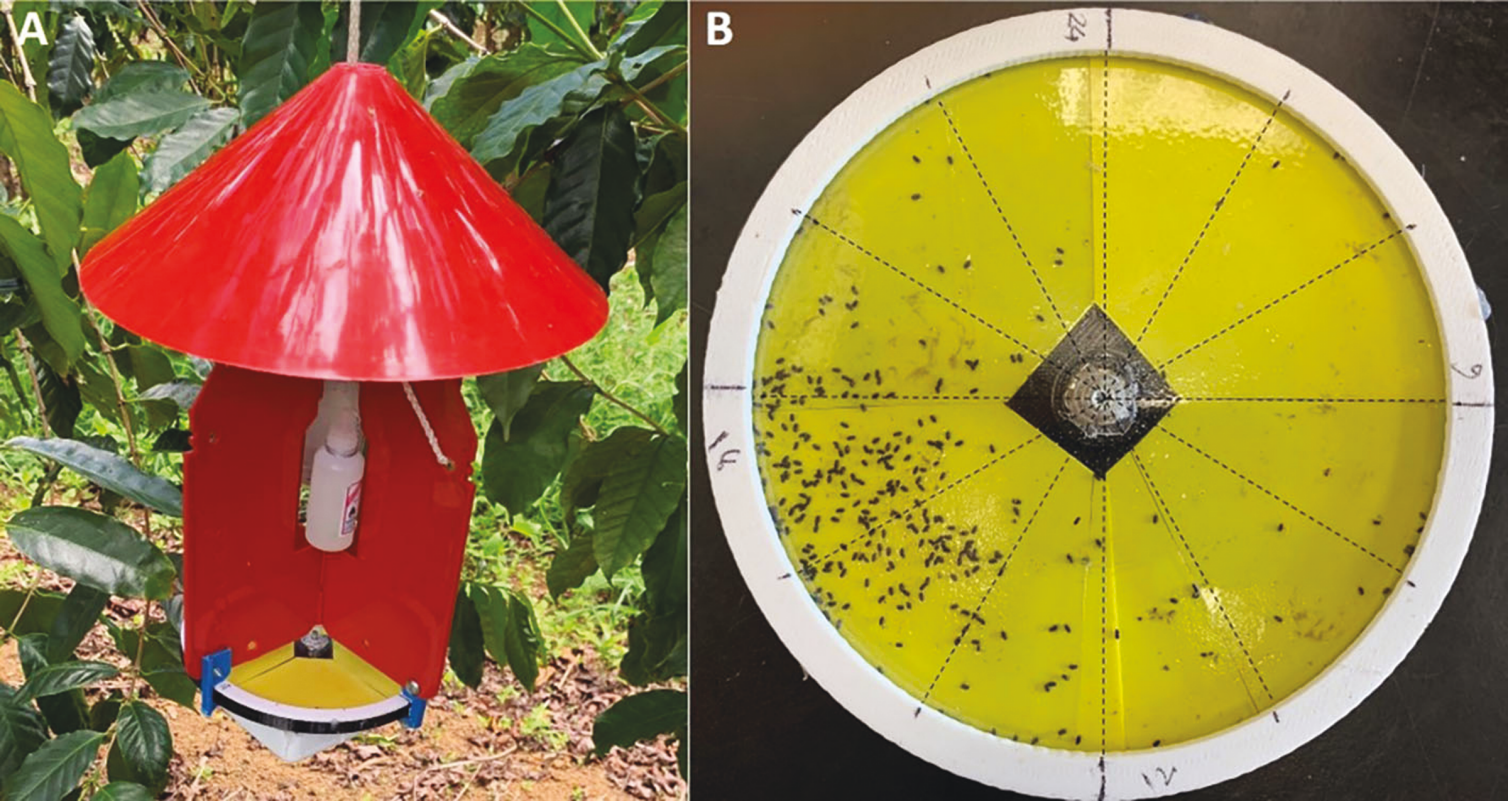
The flight periodicity trap was attached under a Brocap trap and hung in the field A). A representative sample of the sticky dial after being collected from the field shows variability in CBB flight activity B).

To determine the daily flight periodicity of coffee berry borer, a separate experiment was conducted at 4 commercial coffee farms at different elevations on Hawaii Island: Hilo (198 m), Kona (204 m), and Ka’u (393 and 484 m). A novel trap was designed that consisted of (i) a Quartex 24-h high torque quartz clock movement, (ii) a 3D-printed 14 cm round base, (iii) a round yellow sticky dial adhered to the base, (iv) a thin (0.5 mm) transparent plastic disc attached to the hour shaft of the quartz movement, and (v) a 3D-printed holding ring (Fig. 2). At any given moment, the transparent plastic disc exposes an area corresponding to a 30° portion (2-h period) of the sticky dial as it rotates on the hour shaft, gradually exposing the entire sticky dial over a 24-h cycle (Fig. 2A). Because the exposure of the sticky dial occurs gradually rather than abruptly, the recorded flight activity period can overlap by 1 h to either side of the 2-h time frame. The entire arrangement was attached under a Brocap trap after removing the funnel (Fig. 2B). The periodicity of coffee berry borer flight activity is recorded when the coffee berry borer falls into the exposed part of the sticky dial after being intercepted by Brocap trap, which was baited with a 3:1 methanol:ethanol lure (Fig. 3A). These traps were kept in the field for ~2 wk after which the traps were brought to the laboratory and coffee berry borer trapped in the respective areas corresponding to the 24-h dial were counted (Fig. 3B). Two traps were placed in each field and averaged for each 2-wk sampling period. The trial was run from January to December during the 2022 growing season.

### Weather Variables

Cell-service weather stations were used to measure air temperature (°C), relative humidity (%), and wind speed (m/s) on each farm. Stations were comprised of a 4G remote monitoring station (RX3004-00-01, Onset Computer Corporation, Bourne, MA, USA) equipped with a temperature/Relative Humidity (RH) sensor (S-THB-M002) and a wind speed sensor (S-WSET-B). Sensors were located approximately 3–4 m above the ground on each weather station, depending on the type of sensor. Each station was set to log data every 5 min.

### Data Analysis

All statistical analyses were conducted in R v. 4.2.3 (R Core Team 2018). Trap counts were converted to the mean number of coffee berry borer caught per trap per day and log-transformed (log + 1) prior to analysis. Infestation percentages were converted into proportions and then arcsin was transformed prior to analysis. The assumption of normality for each variable was assessed using quantile-quantile plots and a Shapiro-Wilks test; an *F* test was conducted to check for equal variances using the *stats* package. Two separate ANOVAs were run to compare (i) the mean number of coffee berry borer/trap/day captured at each height level and (ii) the infestation height in the trees (low, mid, and high canopy). Each was followed up by a Tukey’s test to determine pairwise differences. To characterize daily flight patterns, 5-min values for the 3 weather variables were first averaged across each 2-h time interval (12–1:59 AM, 2–3:59 AM, 4–5:59 AM, 6–7:59 AM, 8–9:59 AM, 10–11:59 AM, 12–1:59 PM, 2–3:59 PM, 4–5:59 PM, 6–7:59 PM, 8–9:59 PM, 10–11:59 PM). The mean number of coffee berry borer captured at each time interval was correlated with the mean temperature, RH, and wind speed using the Spearman correlation coefficient.

## Results

### Flight Height

**Fig. 4.**
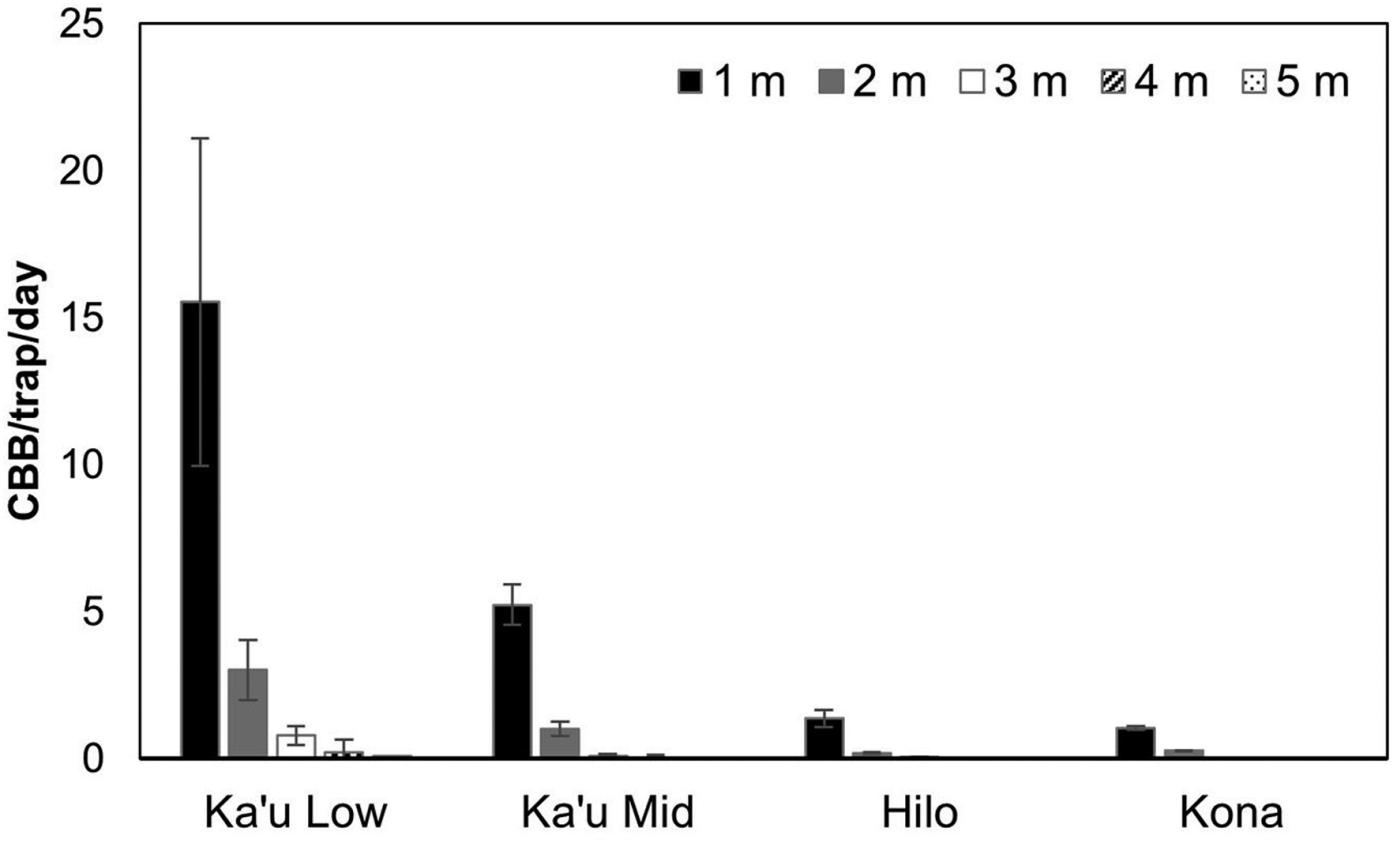
Trap captures (mean ± SEM) of coffee berry borer at 4 farms located on Hawaii Island. Traps were set at 1-m intervals and up to 5 m in height.

**Fig. 5.**
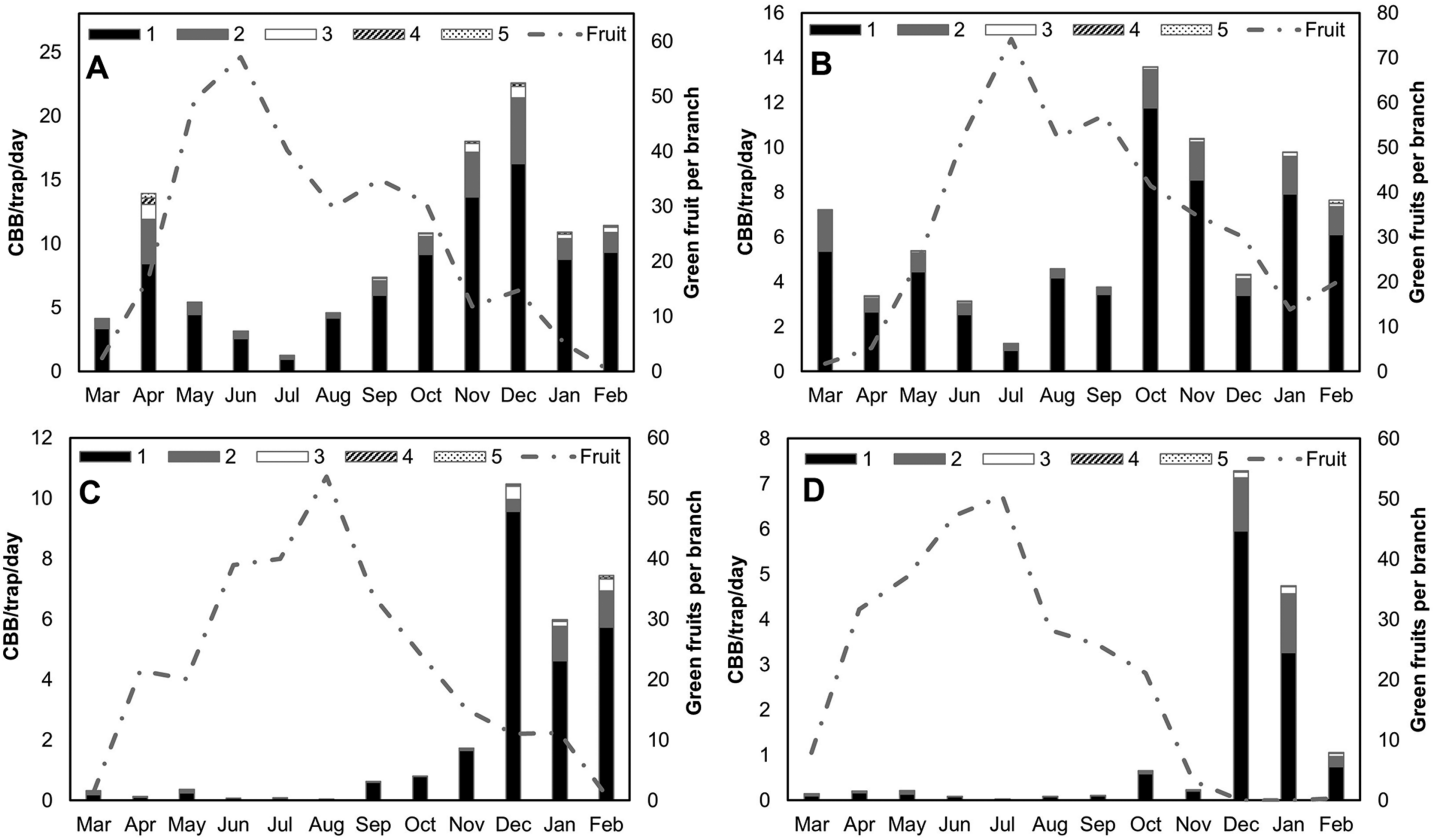
Fruit phenology (dashed line) and trap catch at 5 different heights (1–5 m) in 4 commercial coffee farms on Hawaii Island: Ka’u low A), Ka’u mid B), Hilo C), and Kona D).

Analysis of mean flight height across 4 farms revealed significant differences in coffee berry borer catch among traps (*F* = 130.80, *df* = 4, *P* < 0.001; Fig. 4). The lowest level traps positioned at 1 m caught significantly higher mean numbers of coffee berry borer relative to all other trap heights across the entire season (1 m = 5.79 coffee berry borer/trap/day, 2 m = 1.12 coffee berry borer/trap/day, 3 m = 0.24 coffee berry borer/trap/day, 4 m = 0.06 coffee berry borer/trap/day, 5 m = 0.03 coffee berry borer/trap/day, *P* < 0.001). We observed a consistent pattern of decreasing trap catch with increasing trap height (Fig. 4). This pattern held across farms with varying levels of coffee berry borer activity and throughout the year, even when resources were in decline and more coffee berry borer were flying (e.g. during the harvest and interharvest) (Fig. 5). The only traps that were not significantly different were those at 4 m vs. 5 m (Supplementary Table S1).

**Fig. 6.**
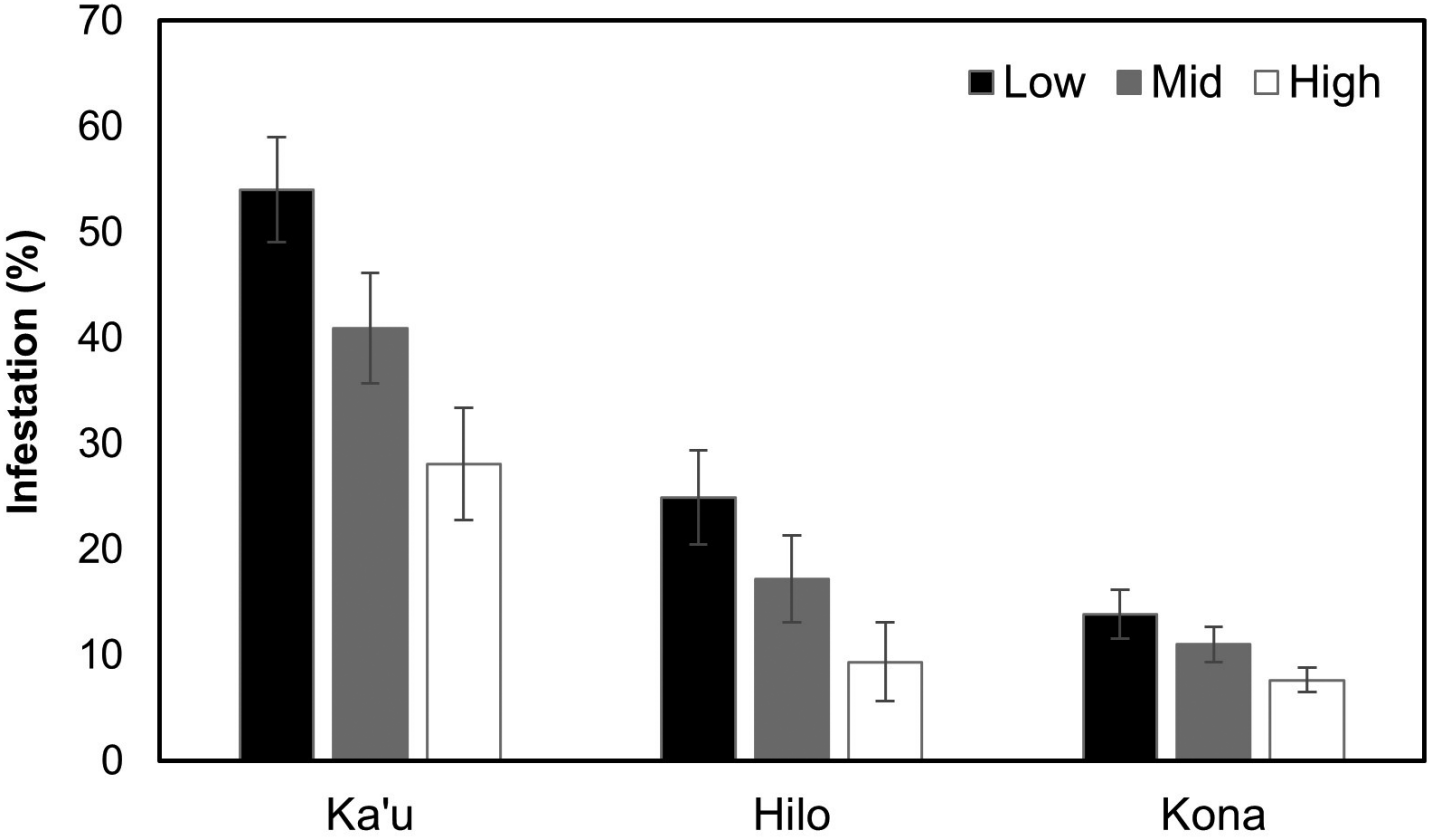
Coffee berry borer infestation (mean ± SEM) on branches located at low (~1 m), mid (~2 m), and high (~3 m) tree canopy levels sampled across 3 commercial coffee farms on Hawaii Island in 2020–2021.

We also observed significant differences in fruit infestation among branches at different canopy heights (*F* = 9.40, *df* = 2, *P* < 0.001; Fig. 6). Infestation on low branches (~1 m high = 35% infestation) was significantly higher than that observed on high-level branches (~3 m high = 17% infestation; *P* < 0.001). No difference in infestation was observed between low and mid branches (~2 m high = 26% infestation; *P* = 0.11) or mid and high branches (*P* = 0.06).

### Daily Flight Periodicity

**Fig. 7.**
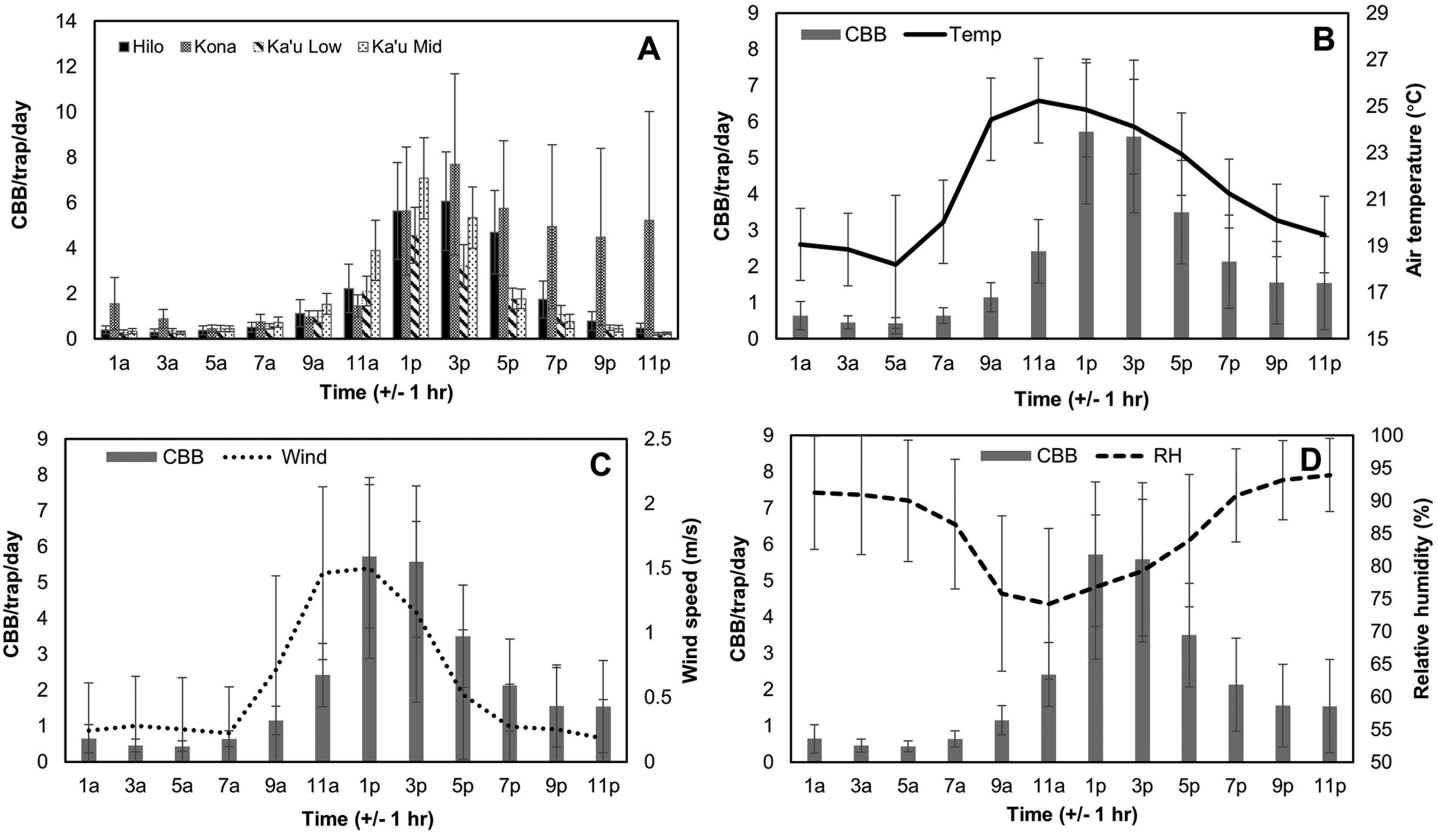
Daily flight periodicity for coffee berry borer across 4 farms on Hawaii Island A). A positive correlation was observed between coffee berry borer flight and air temperature B), as well as wind speed C), while a negative correlation was observed between flight and relative humidity D).

Across all 4 farms examined, coffee berry borer flight peaked in the afternoon hours of 12–4 PM (Fig. 7A). Coffee berry borer flight was minimal in the morning and evening, except for 1 farm in Kona, where the flight was observed to continue at elevated numbers until midnight during the month of March. Daily coffee berry borer flight patterns were found to be positively correlated with air temperature (*r* = 0.46, *P* < 0.001; Fig. 7B) and wind speed (*r* = 0.37, *P* < 0.001; Fig. 7C), but negatively correlated with relative humidity (*r* = –0.40, *P* < 0.001; Fig. 7D). The mean coffee berry borer flight peaked when temperatures were 23–24°C, RH was 76%–79%, and wind speeds were 1.1–1.5 m/s (Fig. 7).

## Discussion

In our examination of the vertical distribution of coffee berry borer, we found that 77%–84% of the total coffee berry borer caught over a 1-yr period were flying at heights of ~1 m. This result coincided with our finding of the highest infestation levels on the lower branches. We observed the same pattern year-round, with no change in flight height or infestation patterns, even as resource availability changed and the numbers of flying coffee berry borer increased. Ruiz-Diaz and Rodrigues (2021) reported a similar phenomenon in Puerto Rico, where 67%–85% of the total coffee berry borer captures were in traps located at 0.5 m above the ground throughout the entire season, and trap catch decreased with increasing height up to 3.5 m. The findings reported in Hawaii and Puerto Rico contrast with a study conducted in Mexico, which reported trap catch at higher altitudes during the interharvest season (Barrera et al. 2005). Garcia-Mendez et al. (2024) posited that the tendency of coffee berry borer to fly higher during the interharvest in Mexico could be to take advantage of the stronger wind currents above the canopy to disperse and locate other resources. In the present study, the tendency of coffee berry borer females to fly at low heights and infest lower branches may be related to several factors. Johnson et al. (2019) reported that many farms examined in Hawaii have a large postharvest reservoir of berries in the trees and on the ground, which may explain why we did not observe coffee berry borer flying above the canopy since there were likely still resources available at lower heights even after the harvest due to poor sanitation practices. Large numbers of coffee berry borer emerge from infested raisins (old berries) that have fallen to the ground after harvest (Johnson et al. 2019). These emerging females likely infest fruit on the lowest branches since they are the first available resources encountered. Lastly, lower wind speeds within the canopy may allow coffee berry borer to better orient to attractants and host-emitted volatiles and thereby locate berries more easily.

Using baited timer traps, we found that the daily coffee berry borer flight peaked from 12 to 4 PM. This finding is in line with that reported for another *Hypothenemus* sp., which began flying at 11 am, peaked at 3 pm, and decreased near sunset (Johnson et al. 2016). A recent study examining flight patterns in coffee berry borer in the shade vs. sun plots reported peak flight times from 2 to 4 PM in the shade and 3–5 pm in the sun (Garcia-Mendez et al. 2024). In contrast, most species of *Xyleborus* ambrosia beetles that have been examined appear to fly closer to dusk when light intensity is low (Menocal et al. 2018). For example, Kendra et al. (2012) reported that *X. glabratus* initiated flight at 4 PM, peaked from 6 to 7 PM, and then declined just before dusk. This suggests that temporal host-seeking flight behavior is species-specific.

We observed that daily coffee berry borer flight increased with increasing air temperature. Like many insects, temperature is a critical factor involved in the dispersal of scolytine beetles (Chen and Seybold et al. 2014, Menocal et al. 2018). At low temperatures, metabolic reactions may be compromised, and the coffee berry borer may be unable to properly activate flight muscles (Pasek 1988). The optimal temperatures reported for other scolytinae species are approximately 20–26 °C (Menocal et al. 2018). In the present study, elevated flight levels were observed when temperatures reached 19 °C, while peak flight coincided with temperatures of ~23–24 °C.

As in several other studies examining bark beetle flight patterns, we found a positive correlation between flight and wind speeds. Chen and Seybold (2014) reported that the walnut borer *Pityophthorus juglandis* Blackman flight was limited at very low wind speeds and peaked when the temperature was ~30 °C and the wind speed was 2 km/h (0.6 m/s). Similarly, Pawson et al. (2017) reported low flight activity of the bark beetle *Hylurgus ligniperda* Fabricius at very low wind speeds, an increase with rising wind speeds, and a peak at 2 m/s. An investigation into the seasonal flight patterns of coffee berry borer in Hawaii by Johnson and Manoukis (2021) reported that flight increased with wind speeds up to 2.5 m/s and declined above that. Our results suggest that coffee berry borer are weak flyers that require some wind to help initiate flight as well as provide olfactory cues to allow detection of resources within the canopy (Srygley and Dudley 2008).

In contrast, a negative relationship was observed between relative humidity and coffee berry borer flight. Coffee berry borer flight tended to peak when the mean daily RH was at its lowest (75%–80%). Bark beetles may not fly during periods of very high RH, as this may indicate rain and an associated drop in barometric pressure, causing them to remain within the berry (Aukema et al. 2005, Chen et al. 2020). In addition, greater coffee berry borer mortality can occur during periods of high RH due to the proliferation of *B. bassiana* under moist, humid conditions (Wraight et al. 2021). Lastly, at very high RH, there is a higher requirement for wing-beat frequency, which is metabolically costly (Krogh and Zeuthen 1941, Klowden 2002).

In the present study, we found that coffee berry borer flight on Hawaii Island primarily occurs at ~1 m in height, with flight decreasing significantly as altitude increases up to 5 m. We observed infestation patterns in the tree that coincided with those observed from trapping; coffee berry borer infestation in low branches (~1 m) was significantly higher than that observed in high branches (~3 m). We also found that daily coffee berry borer flight increased with increasing temperature and wind speed; in contrast, a negative correlation was observed between flight and relative humidity. Across the entire season, peak flight occurred from 12 to 4 PM. Our investigation into coffee berry borer flight activity and infestation suggests that while pesticide sprays should be applied to the whole coffee tree to protect against coffee berry borer and other pests, growers can target low- to mid-level branches at 1–2 m in height to ensure good spray coverage and optimize coffee berry borer mortality. In addition, our examination of flight times suggests that sprays should aim to be conducted in the early to late afternoon when coffee berry borer are actively flying and are most vulnerable to chemical controls.

## Supplementary Material

Supplementary material is available at *Environmental Entomology* online.

nvae051_suppl_Supplementary_Tables_S1
