# Supplementary material for: Vertical and temporal flight patterns of coffee berry borer (Coleoptera: Curculionidae) in Hawaii
Source: Environ Entomol. 2024 May 29;53(4):640–7. doi: 10.1093/ee/nvae051 (PMC11329621; doi:10.1093/ee/nvae051)
Supplement: nvae051_suppl_Supplementary_Tables_S1 [file nvae051_suppl_supplementary_tables_s1.docx]

**Supplementary Materials**

**Table S1.** Results from Tukey’s multiple comparison tests for flight height of coffee berry borer (CBB) based on traps placed at one-meter intervals on coffee farms in Hawaii Island.

| Trap Height (m) | p-value |
| --- | --- |
| 4 vs. 5 | 0.95 |
| 1 vs. 5 | < 0.001 |
| 3 vs. 5 | 0.001 |
| 2 vs. 5 | < 0.001 |
| 1 vs. 4 | < 0.001 |
| 3 vs. 4 | 0.02 |
| 2 vs. 4 | < 0.001 |
| 3 vs. 1 | < 0.001 |
| 2 vs. 1 | < 0.001 |
| 2 vs. 3 | < 0.001 |
